# Delayed Posterior Choroidal Artery Aneurysm Formation and Rupture in a Postsuperior Temporal Artery to Middle Cerebral Artery Bypass Moyamoya Disease Patient

**DOI:** 10.1055/a-2984-6516

**Published:** 2026-11-03

**Authors:** William Wei Hsu, Ryerson Emil Magdaleno Rosario Soliven, Yutaka Igarashi, Takahiro Kanaya, Ryuta Nakae, Shoji Yokobori

**Affiliations:** 1 43989 The George Washington University School of Medicine and Health SciencesWashingtonDistrict of ColumbiaUnited States; 2 Department of Emergency and Critical Care Medicine 26367 Nippon Medical School HospitalBunkyoTokyoJapan

**Keywords:** moyamoya disease, case report, aneurysm, hemorrhage, posterior choroidal artery, bypass, revascularization, STA–MCA

## Abstract

**Background** Moyamoya disease is a rare vascular disease that presents with stenosis of the internal carotid artery accompanied by formation of abnormal, fragile blood vessels at the base of the brain. This pathology often presents with aneurysms, which may cause ischemic or hemorrhagic complications. We present a case of posterior choroidal artery aneurysm in a moyamoya disease patient that ruptured following a 6-year asymptomatic period after the patient received superior temporal artery to middle cerebral artery bypass.

**Observations** A 57-year-old female Japanese patient who received superficial temporal artery to middle cerebral artery bypass for moyamoya disease presented to the Nippon Medical School Hospital emergency room for sudden loss of consciousness after a 6-year asymptomatic period following her bypass procedure. She was diagnosed with left subcortical hemorrhage with ventricular rupture and hydrocephalus associated with a left medial posterior choroidal artery aneurysm. This was then subsequently managed with bilateral ventricular drainage, clipping, spinal drainage, and finally lumbar–peritoneal shunt.

**Conclusion** Our case demonstrates delayed aneurysm formation at the posterior choroidal artery even after bypass surgery. Our patient’s 6-year silent period after her bypass surgery highlights the need for long-term surveillance after the immediate postoperative period even if the patient is asymptomatic due to the possibility of chronic cerebrovascular changes following bypass that may go undetected.

## Introduction

Moyamoya disease (MMD) is a progressive steno-occlusive disease affecting the internal carotid arteries accompanied by the formation of abnormal smoke-like collateral blood vessels at the base of the brain. 1 2 MMD is more common in east Asian countries, with the highest incidence in Japan and being more common in females. 1 One of the common complications of MMD is aneurysm formation with potential rupture and hemorrhaging. 3 4

We present a patient who received superior temporal artery to middle cerebral artery (STA–MCA) bypass surgery for MMD who still developed a posterior choroidal artery (PChA) aneurysm 6 years later. We discuss three other cases of MMD with PChA aneurysm that also received STA–MCA bypass then compare each of their features with those of our own case.

## Illustrative Case

A 57-year-old female Japanese patient who was treated 6 years ago for MMD with STA–MCA bypass presented to the Nippon Medical School Hospital emergency room for sudden loss of consciousness. Computed tomography (CT) scans ( Fig. 1A,B ) indicated she was suffering from left subcortical hemorrhage complicated by hydrocephalus and ventricular rupture. Emergency bilateral external ventricular drainage was performed, and two ventricular drains were inserted to alleviate her elevated intracranial pressure (ICP). The patient was admitted to the intensive care unit at the Nippon Medical School Hospital for further management.

**Fig. 1:**
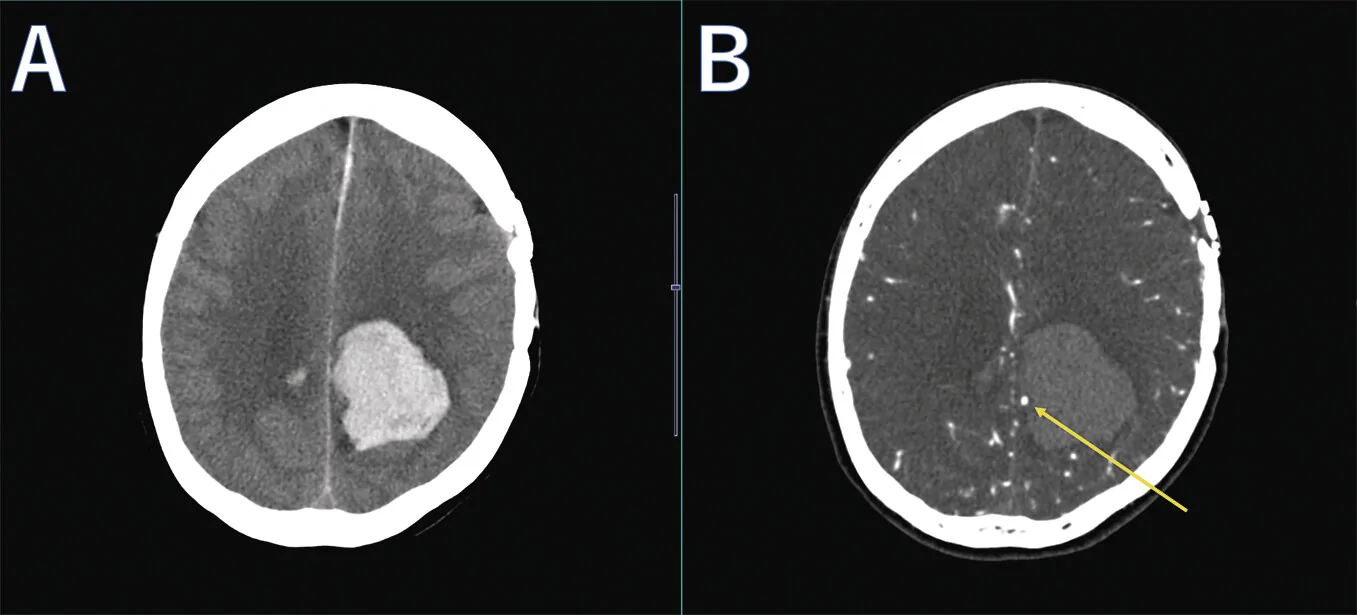
Imaging on admission. ( **A** ) CT angiography demonstrating hemorrhage from ventricular rupture. ( **B** ) CT angiography demonstrating a suspected aneurysm. CT, computed tomography.

Four days after admission, the left ventricular drain was removed as drainage had nearly ceased with little change to the size of the ventricle. The right ventricular drainage had also stopped; however, the drain was left in because it was inserted together with the ICP sensor, which was still needed to continue monitoring pressure. Five days following admission, three-dimensional CT ( Fig. 2A ) angiography and digital subtraction angiography ( Fig. 2B ) were performed, and an aneurysm suggested to be the source of bleeding was found on the left medial PChA. MMD collateral vessels were also observed along with poor anterior circulation.

**Fig. 2:**
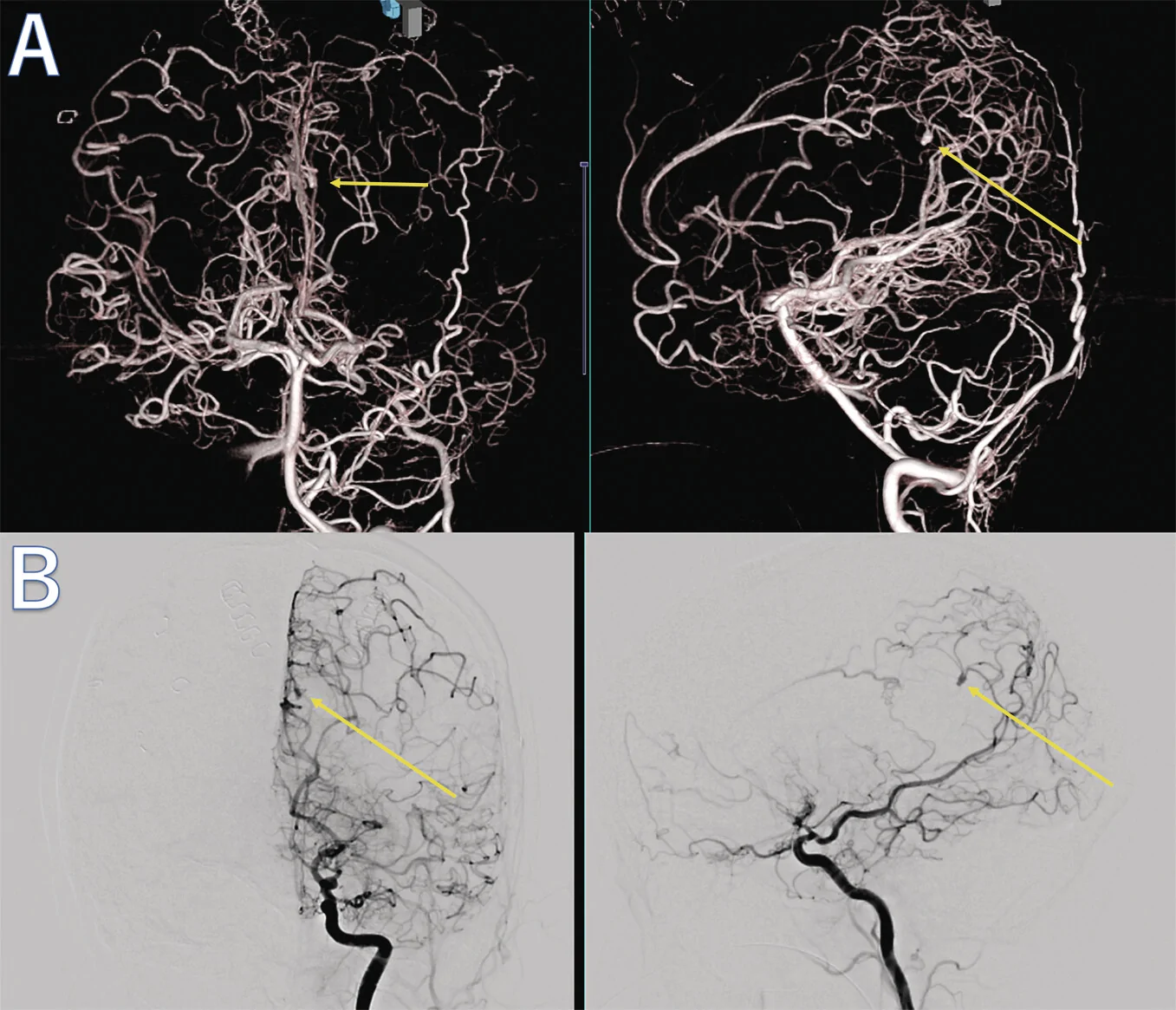
Imaging studies 5 days’ postadmission and ventricular drain insertion demonstrating location of aneurysm in the left medial posterior choroidal artery. ( **A** ) Three-dimensional CT angiography demonstrating the suspected aneurysm site. ( **B** ) Digital subtraction angiography demonstrating the suspected aneurysm site. The classic “puff of smoke” appearance of moyamoya collaterals can also be seen here along with poor anterior circulation. CT, computed tomography.

Two weeks following admission, a clipping procedure was performed that resulted in the aneurysm disappearing with preservation of the parent vessels as seen on cerebral CT scans ( Fig. 3A,B ). The following day, the right ventricular drain and the ICP sensor were removed. CT scans found no new posterior bleeds or ischemic areas; however, the ventricles were slightly enlarged. This was associated with her persistently elevated ICP and prompted a spinal drain to be placed on week 3 for cerebrospinal fluid drainage to lower her ICP.

**Fig. 3:**
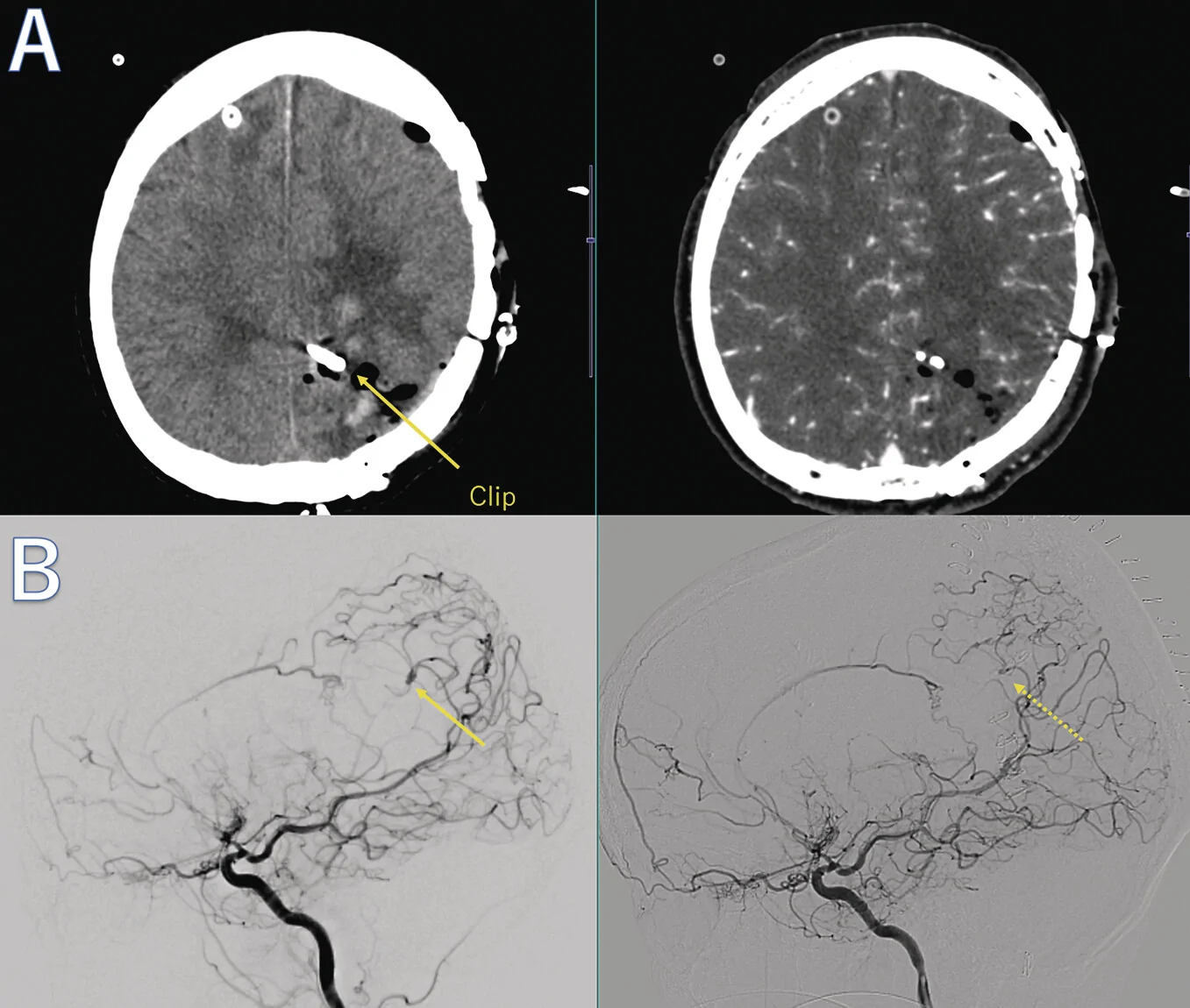
( **A** ) CT angiography immediately after clipping procedure demonstrating the site of the clip. ( **B** ) Digital subtraction angiography showing the site of aneurysm before (left image) and 1 week after (right image) clipping. CT, computed tomography.

At 4 weeks’ postadmission, the spinal drain was removed, and disappearance of the aneurysm was once again confirmed on digital subtraction angiography. At week 5 postadmission, a lumboperitoneal (LP) shunt was created for long-term management of the patient’s elevated ICP. Three days following the LP shunt, a cerebral CT scan was performed and found no significant changes to ventricle size, and her ICP was found to be at an acceptable range with her condition also stabilizing.

## Discussion

### Posterior Choroidal Artery Aneurysm in Moyamoya Disease

MMD-associated aneurysms occur widely throughout the intracranial circulation and are most commonly associated with the posterior circulation. 4 5 6 In addition, aneurysms have been found on the fragile MMD collateral vessels and the distal peripheral vessels such as the anterior choroidal artery and PChA. 7 8 9 10 11 PChA aneurysms in MMD have been theorized to form due to increased blood flow in the posterior circulation to compensate for decreased anterior circulation resulting from stenosis and occlusion of the internal carotid arteries that are characteristic of MMD. 4 8 12 This increased blood flow creates a collateral circulation that increases blood supply to the PChA, causing it to dilate thus increasing hemodynamic stress and increasing the likelihood of aneurysm formation. 4 8 12

From our review of the literature, we have summarized three cases of PChA aneurysm in MMD patients who have also received STA–MCA bypass ( Table 1 ). Among these we found two cases of MMD patients who received bypass following discovery of PChA aneurysms. 8 9 In the case of Suzuki et al, a 51-year-old female presented with minor bleeding from an aneurysm in the PChA. 8 Her aneurysm thrombosed spontaneously 2 months following the onset of bleeding; however, her MMD-associated choroidal anastomoses did not regress, prompting STA–MCA bypass. 8 Following bypass, her anastomoses regressed, and she did not experience recurrence of aneurysms or bleeding as of 2 weeks’ postbypass. In the case of Waga and Tochio, a 6-year-old female with MMD presented with ischemic attacks associated with a lateral PChA aneurysm. 9 She received STA–MCA bypass 3 days later, and the aneurysm was found to have disappeared 6 months following the operation and remained absent 19 months at her last discussed follow-up. 9

**Table 1:** A summary of cases of moyamoya disease patients with posterior choroidal artery aneurysms who received superior temporal artery to medial cerebral artery bypass.

Author and year	Age	Sex	Time of aneurysm formation	Cause of resolution	Rupture
Suzuki et al 2021 8	51	F	Before bypass	Spontaneous Thrombosis	R
Waga and Tochio 1985 9	6	F	Before bypass	STA–MCA	UR
Hashiguchi et al 2024 13	24	F	29-mo postbypass	Improvement in obesity and azilsartan administration	UR
Present case	57	F	6-y postbypass	Clipping	R

Abbreviations: F, female; M, male; R, ruptured; STA–MCA, superior temporal artery to middle cerebral artery; UR, unruptured.

In our literature search, we also found one case of de novo PChA aneurysm formation following STA–MCA bypass surgery. 13 Hashiguchi et al describe a case of an obese 24-year-old female who presented with symptomatic MMD. 13 After being found to have decreased anterior circulation and consequent collateral blood vessels from compensatorily dilated posterior cerebral artery, she received STA–MCA bypass surgery for reperfusion. 13 Postoperatively, her bypass was found to be poorly developed, and 19 months following reperfusion, she was found to have compensatory bilateral dilatation of her PChAs. 13 At 29 months her obesity worsened and she became hypertensive. An aneurysm later formed at the right PChA. 13 After improvements in obesity and lowering of her blood pressure with Azilsartan, the aneurysm disappeared although her PChA dilatation remained. 13 Hashiguchi et al suggest that the poorly developed bypass caused the anterior circulation to continue relying on posterior circulation collaterals to compensate for decreased perfusion. 13 Under continued conditions of hemodynamic instability, her worsening obesity and hypertension were thought to have been the triggers for a PChA aneurysm. 13

### Delayed Postbypass Posterior Choroidal Artery Aneurysm

When compared with the cases of Waga and Tochio and Suzuki et al our case is unique in that despite receiving STA–MCA bypass, our patient experienced aneurysm with rupture after a 6-year silent period. 8 9 However, since neither of these cases were followed long term for more than 6 years, we cannot be sure if either of these cases have also experienced delayed aneurysm formation. 8 9

In contrast, our case is similar to Hashiguchi et al’s case as our case also had a PChA aneurysm following bypass. However, our case also differs in some aspects from Hashiguchi et al’s case which presented with shorter interval of aneurysm formation and obesity-associated etiology with eventual resolution. Our case instead presented with a longer interval, lacked comorbidities, and led to eventual rupture with hemorrhaging. 13 In addition, our lack of prior imaging for our patient made it difficult to determine whether ours was also a case of de novo aneurysm formation.

Due to the discovery of MMD collateral vessels with poor anterior circulation in imaging studies ( Fig. 2A,B ) of our patient, we predict that our case of PChA aneurysm may have resulted from insufficiency of the STA–MCA bypass’s ability to reperfuse the anterior circulation. This makes the etiology of our case most similar to the case of Hashiguchi et al, which was reported to also have a bypass that was not fully developed. 13 Our patient’s lack of comorbidities may explain the longer interval to formation and rupture of the aneurysm as Hashiguchi et al suggest that the aneurysm in their case was induced by short term increases in blood pressure and body mass index while under similar conditions of hemodynamic stress. 13 Furthermore, the 6-year asymptomatic period in our case suggests the possibility of initially sufficient STA–MCA bypass with gradual changes toward insufficiency accompanied by other associated changes such as collateral reformation and eventual appearance of an aneurysm.

### Lessons

When considering cases such as Waga and Tochio, Suzuki et al and our own case where the patient was not monitored following the immediate postoperative period, it is very possible that changes in bypass patency may go undetected until major complications occur. 8 9 We therefore suggest the need for long-term observation even for postbypass patients who are asymptomatic. In accordance with the 2021 Japanese Guidelines for management of MMD we suggest the use of magnetic resonance angiography (MRA) and cerebral angiography (CA) for postoperative evaluation of bypass. 2 Furthermore, we suggest an individualized frequency of imaging during the immediate postoperative period depending on the needs of each individual patient and physician judgment. After recovery, we suggest yearly follow-up imaging with MRA or CA to monitor chronic changes in bypass patency, collateral vessels, and aneurysm formation.

## Conclusion

Our case highlights the potential for delayed aneurysm formation in patients with MMD despite prior surgical revascularization. The development of a PChA aneurysm 6 years after STA–MCA bypass, in the setting of persistent moyamoya collaterals suggests that revascularization may not necessarily eliminate the abnormal collateral hemodynamics associated with MMD. Long-term postoperative vascular surveillance may therefore be warranted to monitor changes in bypass patency, collateral circulation, and aneurysm formation even in the absence of patient symptoms.

## Declaration of GenAI Use

The authors confirm that no Generative AI was used in the preparation of this manuscript.
